# Molecular Characterization of Oncogenic Gene Fusions in a Large Real-World Cohort of Solid Tumors

**DOI:** 10.1158/2767-9764.CRC-25-0329

**Published:** 2025-11-06

**Authors:** Lisa Gai, Bradley Bowles, Adam J. Hockenberry, Brittany Mineo, Christine Chin, Kate Sasser, Halla Nimeiri, Kyle A. Beauchamp, Rotem Ben-Shachar, Justin Guinney, Sandip Pravin Patel, Ben Ho Park

**Affiliations:** 1 Tempus AI, Inc., Chicago, Illinois.; 2 University of California San Diego, La Jolla, California.; 3 Vanderbilt-Ingram Cancer Center, Nashville, Tennessee.

## Abstract

Gene fusions are a class of important oncogenic drivers, with many matched FDA-approved targeted therapies across multiple solid tumors. However, the prevalence of fusions varies considerably by cancer type and assay. Fusion detection is technically challenging, and studies have shown that RNA-based next-generation sequencing (NGS) can improve fusion detection rates when used in conjunction with DNA-based NGS. In this study, we performed a retrospective pan-cancer analysis of 67,278 patients receiving both RNA- and DNA-NGS in 43 distinct solid-tumor cancer types, including non–small cell lung cancer (18.6%), colorectal cancer (18.2%), and breast cancer (13.1%). In this cohort, 1,497 patients (2.2%) had at least one of nine fusions detected—each having an FDA-approved matched therapy in at least one indication. A total of 316 patients (21.1%) had a fusion detected (*RET* or *NTRK1/2/3*) with matched targeted therapy approved in all cancer indications. Concurrent RNA- and DNA-NGS increased the detection of driver gene fusions by 21% compared with DNA-NGS alone. Gene fusions were observed in a range of cancers beyond their approved cancer indications: of 1,501 fusions detected, 29% (*n* = 437) were detected outside of an FDA-approved indication. Finally, emerging fusion drivers with targets in drug development were found in an additional 218 patients, with combined RNA- and DNA-NGS increasing detection of these variants by 127%. Our findings support combined RNA-NGS and DNA-NGS to maximize detection of clinically actionable fusions with FDA-approved matched therapies and potentially actionable fusions in non–FDA-approved indications or those matched to therapies in clinical development.

**Significance:**

This large, real-world pan-cancer study demonstrates that concurrent RNA- and DNA-based NGS significantly improves the detection of clinically actionable gene fusions compared with DNA-NGS alone. Our findings highlight the critical value of integrating RNA-NGS into routine molecular profiling to optimize the detection of driver gene fusions. Doing so may expand the population of patients eligible for matched targeted therapies or clinical trials, particularly in cancers with limited treatment options.

## Introduction

Genomic testing is an important clinical tool to match patients with advanced cancer to optimal therapies (1). Whereas the precise set of clinically actionable genomic alterations differs according to cancer type, gene fusions are an important class of therapeutic targets, with numerous FDA-approved matched therapies associated with strong clinical benefit over standard-of-care therapies (2–6). In contrast to targeted genetic tests or small-scale panels, large genomic panels or whole-transcriptome next-generation sequencing (NGS) assays have the potential to identify a broad set of gene fusions and can accommodate a rapidly changing landscape of fusion-targeting drugs (7–9).

Targeted DNA assays for fusion detection require trade-offs between breadth and depth of coverage, specifically for targeting gene fusions with breakpoints in large introns (5, 10). RNA-NGS provide direct evidence of a fusion expressed at the mRNA level, and detection is not affected by where breakpoints occur (10). In a previous study focused on adenocarcinoma non–small cell lung cancer (NSCLC), the combined use of RNA- and DNA-NGS testing in NSCLC was shown to improve the detection of actionable fusions by 14.3% over DNA-NGS alone (11). However, few, if any, comprehensive analyses have been conducted to evaluate the benefit of RNA-NGS compared with DNA-NGS in detecting multiple fusions with FDA-approved matched therapies across multiple cancers.

Whereas most driver gene fusions are individually rare (<1% prevalence), the combined prevalence of either actionable (FDA-approved matched therapies) or emerging fusions (the subject of current trials or earlier-stage drug development) is large and continues to grow with the addition of new therapies (5, 6, 12). A promising area of treatment expansion is in the use of targeted therapies in a tumor-agnostic manner—therapies that are approved for use in one or more cancer types but are being applied or investigated in cancer types outside of FDA-approved indications (13–17). Understanding the prevalence of specific driver fusions in nonapproved cancer types is important in informing future clinical studies that evaluate targeted therapies in these expanded patient populations (5).

Of the fusion genes that have matched FDA-approved targeted therapy in at least one cancer, only *RET* and *NTRK1/2/3* have FDA approvals that are tumor type–agnostic (5, 18, 19). As molecular diagnostic testing, and RNA-NGS in particular, becomes more widespread across cancer types, there is an opportunity to evaluate the efficacy of therapies in nonindicated cancer types (15). Furthermore, there are studies ongoing to evaluate whether FDA-approved therapies targeting single-nucleotide variants/insertions/deletions can be effective for patients with fusions in the same gene (8).

Here, we describe the molecular landscape of fusion gene drivers across a pan-solid tumor cohort that received concurrent RNA- and DNA-NGS. By assessing the fusion drivers in both FDA-approved and nonapproved indications, we seek to highlight the clinical implications of fusion detection and the potential for expanding therapeutic options in patients with fusions detected in cancer types with limited targeted therapeutic options. Our findings underscore the importance of comprehensive genomic profiling via combined RNA- and DNA-NGS in identifying fusions and highlight the large number of patients harboring potentially actionable fusion drivers in indications without current FDA-approved targeted treatment options.

## Materials and Methods

### Cohort selection

From the deidentified Tempus multimodal database, we selected a cohort of adult patients who received both DNA-NGS (Tempus xT) and RNA-NGS (Tempus xR) from the same tissue-derived biopsy. Briefly, the Tempus database contains structured data derived from direct connections to the electronic health record systems across more than 2,000 institutions, as well as unstructured data collected through technology-enabled chart abstraction and linked NGS results collected during routine clinical care by Tempus AI, Inc.

Patients selected for inclusion in this study were further restricted to only include those with metastatic or stage IV disease, with samples having a minimum of 30% tumor purity (determined via pathology review). If patients had multiple samples, we selected the sample closest to but after metastatic/stage IV diagnosis for analysis. Patients with missing or multiple/conflicting primary diagnoses were excluded, as were patients with hematologic malignancies. Assessed samples derive from a mix of primary and metastatic site biopsies.

### Fusion driver gene selection

**Figure 1.**
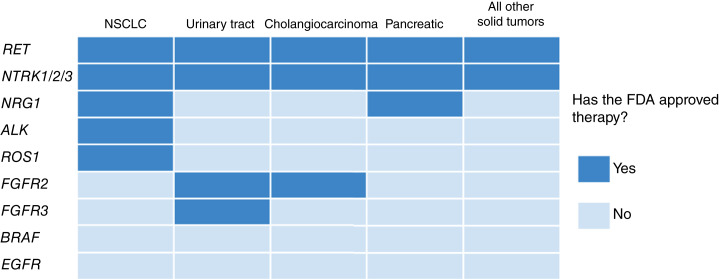
Description of fusions and indications assessed in this study. The grid of fusion drivers (rows) and major indications (columns) highlights the variability in FDA-approved therapies. Given pan-cancer approvals, all listed indications have “indication-matched” approved therapies targeting *RET* and *NTRK1/2/3*. By contrast, *BRAF* and *EGFR* are considered emerging fusion targets as there are no FDA-approved therapies specifically targeting these in any indication. The remainder of assessed fusion drivers have FDA-approved targeted therapies in certain indications but not others (see also Supplementary Table S1).

We determined a set of fusion driver genes to assess based on current FDA approvals. Fusion driver genes for which there is a guideline-recommended therapy in any solid-tumor cancer type were selected for analysis across all cancer types (see Fig. 1; Supplementary Table S1). These actionable fusions included *ALK*, *RET*, *ROS1*, *NTRK1/2/3*, *FGFR2*, *FGFR3*, and *NRG1*. Of these, *NTRK1/2/3* and *RET* have pan-indication approvals whereas other fusion drivers are actionable in specific types. Furthermore, based on emerging evidence and ongoing trials, we separately assessed *BRAF* and *EGFR* fusions across all solid tumors. Whereas there are several other fusion genes with emerging evidence, the nature of our study design required that we limit analyses to genes which were reportable on both our RNA sequencing (RNA-seq) and DNA-seq assays.

### Tissue-based sequencing

All samples were sequenced using the Tempus xT (DNA-NGS, version 4) and Tempus xR (RNA-NGS, version 2) assays. Tempus xT uses DNA isolated from formalin-fixed paraffin-embedded tumor tissue to assess variants in 648 genes with 500× coverage, including single-nucleotide variants, insertions/deletions, copy-number variants, and enhanced detection of select genomic rearrangements (22 genes, which include all fusions assessed here). Tempus xR is a whole-transcriptome RNA-seq assay similarly utilizing formalin-fixed paraffin-embedded tumor tissue. All assessed samples passed quality control metrics for both DNA-NGS and RNA-NGS.

Details on the Tempus DNA fusion detection pipeline have been previously published (20, 21). Briefly, this assay identifies structural variants (SV) in a set of 22 enhanced gene targets (so noted because the assay uses both exonic and select intronic probes) through alignment to the human genome (GRCh37). SVs are called by LUMPY3, with filters based on supporting read count for quality control and annotated by an enhanced version of AGFusion4.

Details on the Tempus xR fusion detection pipeline have been previously published (22). Briefly, this assay uses an ensemble method that integrates two SV detection algorithms (STAR-Fusion and Mojo), filters putative fusions by accounting for technical and biological noise, and annotates candidate fusions with an enhanced version of AGFusion. Gene expression transcript per million (TPM) values are generated using Kallisto as previously described (22, 23).

Tumor mutational burden (TMB) was calculated, based off of Tempus xT results, by dividing the number of nonsynonymous mutations by the megabase size of the panel. All nonsilent somatic coding mutations, including missense, insertions/deletions, and stop-loss variants with coverage greater than 100× and an allelic fraction greater than 5%, were included. TMB-high samples were defined as those having ≥10 mutations/megabase.

### xR analytic validation–limit of blank

The limit of blank (LOB) for the Tempus xR RNA-seq assay was determined by establishing the threshold of total supporting reads at which a negative call is confidently called. Methods and samples are described in Supplementary Text S1 and Supplementary Table S2. In brief, 24 unique samples expected to be wild-type for gene fusions across a range of cancer types were tested in duplicate using two different reagent lots (*n* = 4 per sample) for a total of 96 measurements. All samples other than one glioblastoma sample had zero total supporting reads detected for reportable fusions across all lot numbers. The glioblastoma sample had three total supporting reads for fusion *VOPP1*–*EGFR*. The LOB threshold was set at three total supporting reads for fusions with ≥4 total reads required to call a positive fusion.

### xR analytic validation–fusion accuracy

The accuracy of Tempus xR fusion calling was evaluated by comparing the xR fusion detection results with the FusionPlex BBI Solid Tumor Panel performed at the University of Washington Clinical Genomics Laboratory, a College of American Pathologists/Clinical Laboratory Improvement Amendments–certified laboratory. A total of 290 unique samples were evaluated for variant-level concordance across a range of cancer types (see Supplementary Tables S3 and S4 for more details). There was one false positive and three false negatives. The positive percent agreement was 98.2% (95% confidence interval, 94.97%–99.40%) and negatigve percent agreement was 99.993% (95% confidence interval, 99.96%, ≥99.99%).

### Fusion classification by assay

The Tempus xT (DNA-NGS) and xR (RNA-NGS) pipelines are run separately and manually reviewed as part of the Tempus clinical workflow, a process that includes assessing fusion driver and partner, support in RNA-NGS and DNA-NGS, and the presence of an intact protein domain that, when activated, can result in abnormal cell proliferation to determine whether the fusion should be included in a clinical report. For this study, we used the following research-only classification system to determine whether a particular reported fusion in a single patient sample was detected by RNA- or DNA-NGS. A fusion was considered detected by RNA-NGS if it had a read support above the LOB. A fusion was considered detected by DNA-NGS if it had a read support of at least 15 if a matching fusion partner was detected in RNA-NGS or a read support of at least 35 regardless of RNA-NGS support. These research thresholds were selected based on prior analytic validation and studies to balance sensitivity and specificity and minimize false positives in clinical reporting (11). In all cases in which a specific partner was required for an analysis and when bioinformatic evidence showed support for multiple fusion partners in RNA-NGS, we selected the partner with breakpoints that keep the relevant domain intact and with the highest support. If multiple partners were detected via DNA-NGS, we selected the partner with the highest read support. Driver fusions were classified as being detected by both DNA-NGS and RNA-NGS on a per-patient level, regardless of whether the detected fusion partners and/or breakpoints were identical.

### Statistical analysis

Differences in demographics and clinical features between fusion-positive and fusion-negative patients were assessed via *χ*^2^ test for categorical values and Mann–Whitney U test for quantitative values. Differences in fusion partners detected by RNA-NGS were also assessed via *χ*^2^ test. Differences in RNA expression between fusion-positive and fusion-negative patients were assessed via Mann–Whitney U test for individual cancer types. To assess the overall effect of fusion status (positive or negative) on observed expression, the lmerTest R package was used to generate a random effects model of expression TPM with cancer type as the random effect (24). The Satterthwaite degrees of freedom method was used to evaluate the significance of the fusion status on expression TPM.

### Data and research ethics

This study was conducted on deidentified health information subject to an Institutional Review Board–exempt determination (Advarra Pro00072742) and did not involve human subjects’ research.

## Results

### Fusion prevalence by detection method

The cohort used to assess fusion prevalence consisted of 67,278 deidentified patient records with advanced solid tumors who underwent successful concurrent RNA-NGS and DNA-NGS testing. The median (IQR) age was 65.7 (57.4–73.2) years, 33,109 patients (49.2%) were female, and 30,985 patients (46.1%) were known current or former smokers (Table 1). The cohort was comprised of 43 cancer types, with NSCLC as the most prevalent with 12,518 patients (18.6% of cohort) followed by colorectal cancer with 12,211 (18.2%; Supplementary Table S5).

**Table 1.** Cohort demographics and clinical features.

​	All (*n* = 67,278)	Fusion-positive (*n* = 1,497)	Fusion-negative (*n* = 65,781)
Age at biopsy (*P* < 0.001)	​	​	​
Median (25%, 75%)	65.65 (57.42, 73.2)	63.31 (53.36, 71.26)	65.7 (57.51, 73.24)
Tumor purity (*P* = 0.02)	​	​	​
Mean (std)	57.58 (17.11)	56.51 (16.92)	57.6 (17.12)
Gender (*P* < 0.001)	​	​	​
Male (*n*)	50.8% (34,169)	45.8% (686)	50.9% (33,483)
Female (*n*)	49.2% (33,109)	54.2% (811)	49.1% (32,298)
Race (*P* = 0.001)	​	​	​
White (*n*)	49.9% (33,595)	48.9% (732)	50.0% (32,863)
Black or African American (*n*)	7.7% (5,190)	6.6% (99)	7.7% (5,091)
Asian (*n*)	2.5% (1,663)	4.1% (61)	2.4% (1,602)
Race not stated (*n*)	0.6% (416)	0.7% (11)	0.6% (405)
Other (*n*)	3.9% (2,605)	4.5% (67)	3.9% (2,538)
Unknown (*n*)	35.4% (23,809)	35.2% (527)	35.4% (23,282)
Smoking status (*P* < 0.001)	​	​	​
Current or former smoker (*n*)	46.1% (30,985)	36.2% (542)	46.3% (30,443)
Nonsmoker (*n*)	34.5% (23,235)	45.2% (676)	34.3% (22,559)
Unknown (*n*)	19.4% (13,058)	18.6% (279)	19.4% (12,779)

*P* values result from comparisons between fusion-positive and fusion-negative patients using the relevant statistical test.

**Figure 2.**
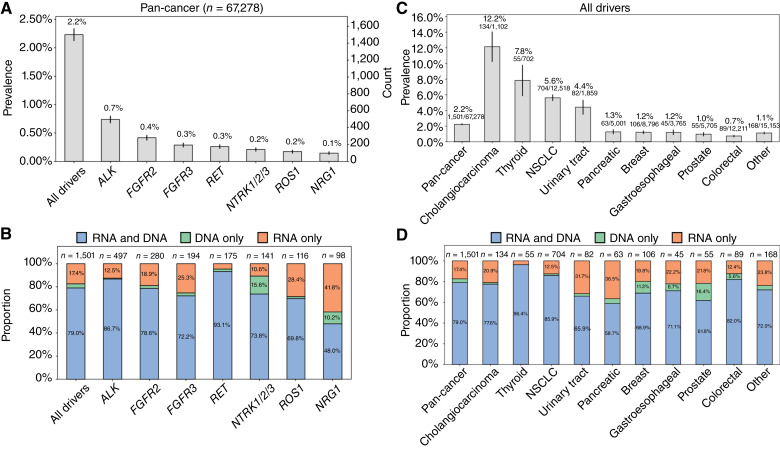
Fusion detection by analyte and cancer type. **A,** Pan-cancer fusion prevalence by fusion driver. **B,** For each fusion driver, percent of fusions detected by analyte: RNA-only, DNA-only, or detected by both RNA and DNA. **C,** Combined fusion prevalence by cancer type for all nine driver fusions. **D,** For each cancer type, percent of combined driver fusions detected by analyte: RNA-only, DNA-only, or detected by both RNA and DNA. For **C** and **D**, cancer types with <500 patients were aggregated into the “other” category. Four patients had multiple fusions detected: *FGFR2* and *ROS1*, *NRG1* and *NTRK3*, *NRG1* and *RET*, and *RET* and *ROS1*.

A total of 1,501 distinct fusions in at least one of the nine driver genes assessed (*ALK*, *RET*, *ROS1*, *NTRK1/2/3*, *FGFR2*, *FGFR3*, and *NRG1*; Fig. 1) were detected in 1,497 patients (Table 1; Fig. 2) for a combined prevalence of 2.2% across the pan-cancer cohort. Among the assessed demographic and clinical features, age (Mann–Whitney U test; *P* < 0.001), tumor purity (Mann–Whitney U test; *P* = 0.02), race (*χ*^2^ test, *P* = 0.025), gender (*χ*^2^ test, *P* < 0.001), and smoking status (*χ*^2^ test, *P* < 0.001) differed significantly between the fusion-positive and fusion-negative groups (Table 1). Fusion-positive patients were specifically younger and more likely to be females and nonsmokers. Given the possible confounding effects of performing a combined analysis of cancer types with their own distinct patient characteristics, we separately performed these analyses for the two largest cohorts in our dataset: NSCLC (Supplementary Table S6) and colorectal cancer (Supplementary Table S7). Consistent with other literature, NSCLC fusion-positive patients were younger and were more likely to be nonsmokers than fusion-negative patients, whereas in patients with colorectal cancer, fusion-positive patients were older than fusion-negative patients and there was no difference in smoking status.

The prevalence of individual driver fusions varied (Fig. 2A), with *ALK* fusions having the highest at 0.7% (*n* = 497) and *NRG1* fusions the lowest at 0.15% (*n* = 98). Notably, RNA-NGS substantially improved the detection of all fusions, with 17% of patients harboring a fusion that was detected solely by RNA-NGS (Fig. 2B)—a 21% increase in patients with a detected fusion due to concurrent RNA- and DNA-NGS compared with DNA-NGS alone. RNA-NGS increased fusion detection for all driver genes relative to DNA-NGS alone, ranging from 2.3% of *RET* fusions to 41.8% of *NRG1* fusions being detected only via RNA-NGS (Fig. 2B). Fusion details, including partners detected by analyte (RNA-NGS and/or DNA-NGS) as well as corresponding read support statistics, are provided in Supplementary Tables S8 and S9.

When assessing individual cancer types, the combined prevalence of the nine driver fusions varied from 12.2% (*n* = 134/1,102) for cholangiocarcinoma to 0.7% (*n* = 89/12,211) for colorectal cancer (Fig. 2C). Among the nine cancer types with the highest fusion counts, the percentage of fusions detected by RNA-NGS alone ranged from 3.6% (*n* = 2/55) for thyroid cancer to 36.5% (*n* = 23/63) for pancreatic cancer (Fig. 2D).

A previous study of patients with NSCLC showed that fusion drivers detected by RNA-NGS alone were enriched in patients with low TMB (25). Overall, among TMB-low patients, the percentage of patients with a driver fusion (2.4%, 1,369/57,399) was significantly higher than in TMB-high patients (1.3%, 127/9,835, *χ*^2^-test, *P* < 0.001). In total, 91.5% of patients (*n* = 1,369/1,497) with a driver fusion were TMB-low; similarly, the majority of patients with RNA-only fusions were TMB-low (88.9%, *n* = 232/269), suggesting that in fusion-positive patients, the fusion is the primary oncogenic driver (Supplementary Table S10).

### Fusion detection in FDA-approved and nonapproved indications

**Figure 3.**
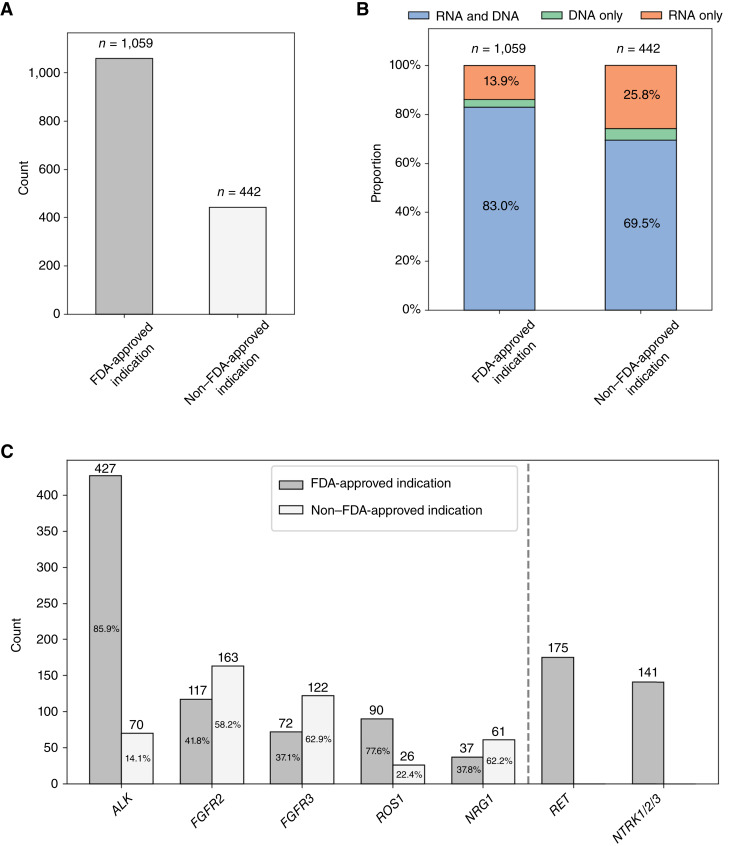
Fusion detection stratified by cancer indications with and without FDA-approved targeted therapy. **A,** Fusions detected in FDA-approved and non–FDA-approved indications aggregated across fusion drivers (see Supplementary Table S1). **B,** Percent of driver fusions detected by analyte for FDA-approved and non–FDA-approved indications. **C,** Counts of fusions detected in FDA-approved and non–FDA-approved indications by fusion driver.

Only *NTRK1/2/3* and *RET* fusions are FDA-approved for indication-agnostic matched therapies. We therefore investigated the rate of fusions detected in FDA-approved and nonapproved matched indications. Of the 1,501 fusions detected, 1,059 (70.6%) of fusions were detected in FDA-approved indications (Fig. 3A). Overall, combined RNA- and DNA-NGS increased the number of patients with a detected actionable fusion by 16% relative to DNA-NGS alone (Fig. 3B). By contrast, 442 fusions (29.4%) were detected in the nonapproved indications (Fig. 3A), with combined RNA- and DNA-NGS improving the detection of these variants by 35.3%—an addition of 114 fusions detected only via RNA-NGS (Fig. 3B). The difference in the proportion of fusions detected only via RNA-NGS versus those detected by DNA-NGS was statistically significant between the FDA-approved and nonapproved indications (*χ*^2^ test: *P* < 0.001). Variability in FDA-approved versus nonapproved indications was observed for specific fusions, with 85.9% of all *ALK* fusions detected in the FDA-approved indication of NSCLC (Supplementary Table S11). By contrast, for *FGFR2*, *FGFR3*, and *NRG1*, more than half of the detected fusions occurred in nonapproved indications (Fig. 3C). Overall, 13.6% and 8.2% of *FGFR2* fusions were in nonapproved breast and pancreatic cancer indications, respectively, 22.2% of *FGFR3* fusions were in nonapproved NSCLC, and 19.4% and 10.2% of *NRG1* fusions were in nonapproved breast and prostate cancers, respectively (Supplementary Table S11).

**Figure 4.**
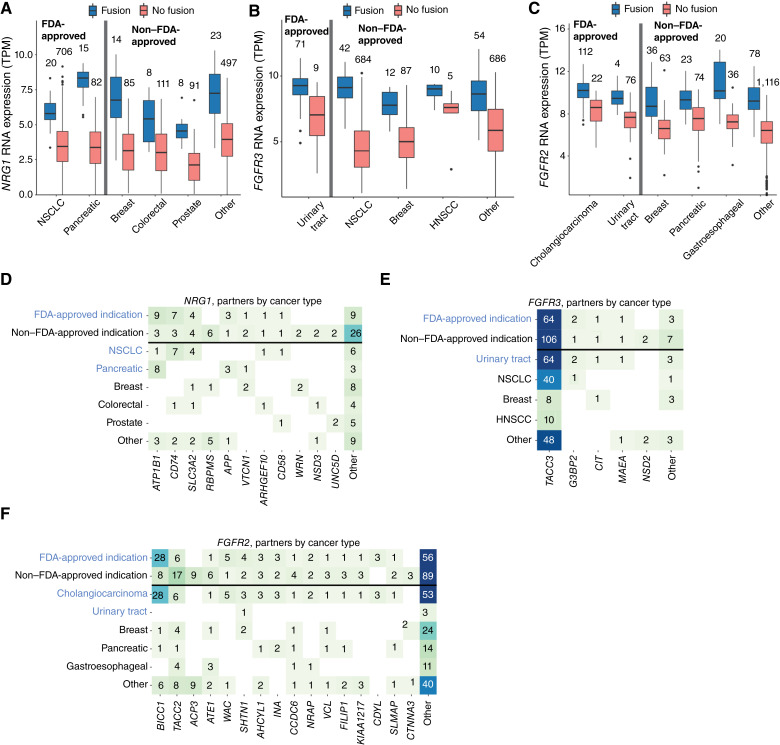
Gene expression and RNA fusion partners in *FGFR2*, *FGFR3*, and *NRG1* fusions. **A–C,** Gene expression TPM by cancer type in fusion-positive and fusion-negative patients for (**A**) *NRG1*, (**B**) *FGFR3*, and (**C**) *FGFR2*. All FDA-approved indications and non–FDA-approved indications are separated by the gray lines. **D–F,** Fusion driver partners identified by RNA stratified by cancer type for the following fusions: (**D**) *NRG1*, (**E**) *FGFR3*, and (**F**) *FGFR2*. All FDA-approved indications and the top three non–FDA-approved indications by fusion count are shown, with the remaining non–FDA-approved indications grouped into the “other” cancer type category. Fusion partners that only appear in a single patient (or only in two patients in the case of *FGFR2*) are grouped into the “other” partner category. HNSCC, head and neck squamous cell carcinoma.

We hypothesized that these fusions—*FGFR2/3* and *NRG1*—may function as oncogenic drivers in the nonapproved indications and therefore could be candidates for future drug approval studies. To test this hypothesis, we assessed differences in gene expression between fusion-positive and fusion-negative samples in both FDA-approved and nonapproved indications (Fig. 4A–C). Within each cancer type, we observed that the fusion-positive samples had significantly higher gene expression than the fusion-negative samples in both FDA-approved and nonapproved indications (*P* < 0.001 for each fusion).

Next, we compared fusion partner genes in FDA-approved versus nonapproved indications for *FGFR2*, *FGFR3*, and *NRG1* fusions (Fig. 4D–F). For *FGFR2*, *BICC1* was the most common partner gene observed in FDA-approved indications whereas *TACC3* was the most common partner gene in nonapproved indications. Notably, we observed nine instances of *ACP3*–*FGFR2* fusions in patients with prostate cancer, which were the only observed instances of this fusion pairing across our cohort. Partners differed significantly between approved and nonapproved indications (*χ*^2^ test, *P* = 0.03) for *FGFR2*. In *FGFR3* driver gene fusions, *TACC3* was the most abundant fusion partner regardless of indication, with only rare instances of other fusion partners observed. Finally, *NRG1* fusion partners were most commonly *ATP1B1* (pancreatic cancer) or *CD74* (NSCLC) in FDA-approved indications, with a mix of these as well as *SLC3A2* and *RBPMS* being commonly observed partner genes in nonapproved indications. Partners did not differ significantly between approved and nonapproved indications for *FGFR3* or *NRG1* (*χ*^2^ test, *P* > 0.05 for both).

### Emerging fusions

Finally, we assessed two fusions (which are reportable on both the DNA- and RNA-seq assays) that have targeted therapies in clinical development but with no FDA-approved drugs in any indications: *BRAF* and *EGFR*. Among the demographic and clinical features, gender (*χ*^2^ test, *P* < 0.001), smoking status (*χ*^2^ test, *P* < 0.001), and indication (*P* < 0.001) were the only features that differed significantly between emerging fusion-positive and emerging fusion-negative patients (Supplementary Tables S12 and S13).

**Figure 5.**
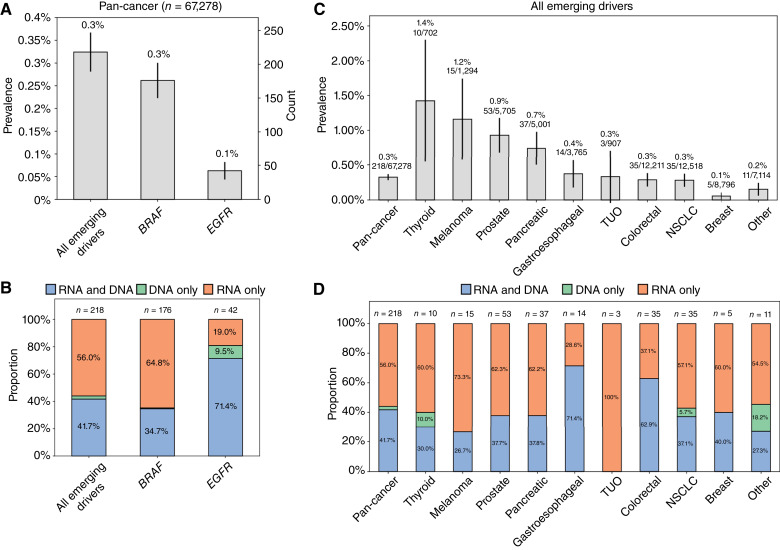
Emerging fusion detection by analyte and cancer type. **A,** Pan-cancer fusion prevalence by emerging fusion driver. **B,** For each emerging fusion driver, percent of fusions detected by analyte: RNA-only, DNA-only, or detected by both RNA and DNA (all patients received both RNA-NGS and DNA-NGS testing). **C,** Combined emerging fusion prevalence by cancer type for the two emerging driver fusions. **D,** For each cancer type, percent of combined emerging driver fusions detected by analyte: RNA-only, DNA-only, or detected by both RNA and DNA. For **C** and **D**, cancer types with <500 patients were aggregated into the “other” category. TUO, tumor of unknown origin.

We identified 218 patients with *BRAF* (*n* = 176) or *EGFR* (*n* = 42) fusions (Fig. 5A), with a combined prevalence of 0.3% across all solid tumors. Fusions detected only by RNA-NGS accounted for 56% (*n* = 122) of the total, 64.8% (*n* = 114) of *BRAF* fusions and 19.0% (*n* = 8) of *EGFR* fusions (Fig. 5B). Concurrent RNA- and DNA-NGS increased the detection of these emerging fusions by 127% relative to DNA-NGS alone. The combined prevalence of these two emerging fusion drivers was typically <1% for each cancer type, with thyroid and melanoma cancers being notable exceptions with prevalences of 1.4% and 1.1%, respectively (Fig. 5C). In these cancer types, the inclusion of RNA-NGS testing in addition to DNA-NGS increased the number of emerging fusions detected by 150% for thyroid cancer (six fusions detected only via RNA-NGS vs. four detected by DNA-NGS or both assays) and 275% for melanoma (11 detected only via RNA-NGS vs. 4 detected by DNA-NGS; Fig. 5D). Supplementary Fig. S1 presents these analyses separately for *EGFR* and *BRAF* fusions.

## Discussion

In a large, diverse, real-world cohort of advanced, pan-cancer patients, we assessed the prevalence of nine fusions—each with an FDA-approved matched therapy in at least one indication—and found that concurrent RNA-NGS and DNA-NGS improves fusion detection by 21% compared with DNA-NGS alone. The improvement in detection rate via RNA-NGS testing was higher for fusions detected in non–FDA-approved indications than in FDA-approved indications (35.5% vs. 16%, respectively). In two further emerging fusions—each having a targeted therapy in clinical development—concurrent DNA- and RNA-NGS more than doubled fusion detection compared with DNA-NGS alone, largely driven by *BRAF* fusions.

The fusion gene drivers that showed the highest improvement in detection rate via RNA-NGS (*NRG1* and *BRAF*) both have large intronic regions which may make them challenging to detect with targeted DNA-NGS panels (26–28). Along with findings that fusion partners detected via RNA-NGS vary widely by cancer type, our study underscores the complexity in developing pan-cancer targeted DNA-NGS assays that need to balance assay sensitivity and read coverage. By contrast, whole-transcriptome RNA-NGS can detect new and rare fusion partners and provide direct evidence that a rearrangement produces an expressed fusion. This may be particularly important in less–well-characterized fusions occurring in non–FDA-approved indications, as evidenced by the large number of fusions detected solely by RNA-NGS in non–FDA-approved indications compared with FDA-approved indications. Taken together with the higher fusion detection rates we observed via concurrent RNA- and DNA-NGS, these findings demonstrate that RNA-NGS should be used routinely to maximize fusion detection.

Basket trials have shown the efficacy of therapies targeting fusion drivers across solid tumors, leading to pan-cancer approvals for *RET* (18, 29, 30) and *NTRK1/2/3* fusions (31). These studies are notable as they increase the number of fusion-positive patients that can benefit from targeted therapy compared with cancer-specific studies. In our study, more patients had *NRG1*, *FGFR2*, and *FGFR3* fusions detected in non–FDA-approved indications than in FDA-approved indications. Notably, the most common non–FDA-approved indications were cancers with limited targeted therapies available: pancreatic cancer for *FGFR2* fusions (prevalence of 0.5%) and prostate cancer for *FGFR3* and *NRG1* fusions (combined prevalence of 0.4%). Even when assessing *ALK* fusions, the most routinely screened for fusion in NSCLC, 14% of *ALK* fusions were detected outside of NSCLC.

Widespread adoption of RNA-NGS testing in future studies may expand the eligible patient population for receiving these drugs or enrolling in clinical trials, ultimately increasing access to targeted therapy options if studies are successful. This was evident in the registrational phase II eNRGy trial of patients with advanced *NRG1* fusion-positive cancer in any tumor type that received zenocutuzumab (32). In this study, most fusion-positive patients were identified by RNA-NGS, and the study resulted in the recent FDA approval of zenocutuzumab in December 2024 for patients with NSCLC and pancreatic adenocarcinoma harboring *NRG1* fusions (33).

Our study has several limitations. Fusion detection was assessed via a single, commercial NGS platform. Detection rates may vary among commercial platforms, though the results of this study are comparable with other published studies (34–37). Additionally, our study does not account for potential differences in assay failure rates between RNA-NGS and DNA-NGS (38). Many factors can affect RNA-NGS failure rates, such as time from biopsy to sequencing or low tumor purity (39), with dramatic differences in failure rate seen between studies depending on the criteria for sample inclusion (40, 41). Instead, we focus on patients that received successful RNA-NGS and DNA-NGS to directly compare fusion detection rates.

### Conclusion

To our knowledge, this is the largest retrospective study of an advanced, pan-cancer cohort of real-world patients tested with concurrent RNA-NGS and DNA-NGS that quantifies the prevalence of fusions with FDA-approved targeted therapies and emerging fusions. Our findings demonstrate that concurrent RNA-NGS and DNA-NGS maximizes fusion detection across cancer types and has the potential to expand access to matched targeted therapies for patients.

## Supplementary Material

Supplementary Text Supplementary Text S1

Supplementary Figure 1 Supplemental Figure 1

Supplementary Table 1 Supplemental Table 1

Supplementary Table 2 Supplemental Table 2

Supplementary Table 3 Supplemental Table 3

Supplementary Table 4 Supplemental Table 4

Supplementary Table 5 Supplemental Table 5

Supplementary Table 6 Supplemental Table 6

Supplementary Table 7 Supplemental Table 7

Supplementary Table 8 Supplemental Table 8

Supplementary Table 9 Supplemental Table 9

Supplementary Table 10 Supplemental Table 10

Supplementary Table 11 Supplemental Table 11

Supplementary Table 12 Supplemental Table 12

Supplementary Table 13 Supplemental Table 13

## Data Availability

Deidentified data used in the research were collected in a real-world health care setting and are subject to controlled access for privacy and proprietary reasons. When possible, derived data supporting the findings of this study have been made available within the article and its Supplementary Figures/Tables. Further details may be made available through the corresponding author upon reasonable request.

## References

[1] Mosele MF , Westphalen CB, Stenzinger A, Barlesi F, Bayle A, Bièche I, . Recommendations for the use of next-generation sequencing (NGS) for patients with advanced cancer in 2024: a report from the ESMO Precision Medicine Working Group. Ann Oncol 2024;35:588–606. 38834388 10.1016/j.annonc.2024.04.005

[2] Nikanjam M , Okamura R, Barkauskas DA, Kurzrock R. Targeting fusions for improved outcomes in oncology treatment. Cancer 2020;126:1315–21. 31794076 10.1002/cncr.32649 PMC7050395

[3] Morand S , Rager L, Craig D, Nemunaitis A, Choucair K, Rao D, . Clinical characterization and therapeutic targeting of fusion genes in oncology. Future Oncol 2025;21:1249–60. 40128124 10.1080/14796694.2025.2477974 PMC11988278

[4] Zhou C , Solomon B, Loong HH, Park K, Pérol M, Arriola E, . First-line selpercatinib or chemotherapy and pembrolizumab in RET fusion-positive NSCLC. N Engl J Med 2023;389:1839–50. 37870973 10.1056/NEJMoa2309457 PMC10698285

[5] Ahmed J , Torrado C, Chelariu A, Kim S-H, Ahnert JR. Fusion challenges in solid tumors: shaping the landscape of cancer care in precision medicine. JCO Precis Oncol 2024;8:e2400038. 38986029 10.1200/PO.24.00038 PMC11371109

[6] Schram AM , Chang MT, Jonsson P, Drilon A. Fusions in solid tumours: diagnostic strategies, targeted therapy, and acquired resistance. Nat Rev Clin Oncol 2017;14:735–48. 28857077 10.1038/nrclinonc.2017.127 PMC10452928

[7] Bhamidipati D , Pellatt A, Subbiah V. Targeting all BRAF alterations: the (Re)-search continues. JCO Precis Oncol 2024;8:e2300670. 38380848 10.1200/PO.23.00670 PMC10896466

[8] Liu SV , Nagasaka M, Atz J, Solca F, Müllauer L. Oncogenic gene fusions in cancer: from biology to therapy. Signal Transduct Target Ther 2025;10:111. 40223139 10.1038/s41392-025-02161-7 PMC11994825

[9] Gao Q , Liang W-W, Foltz SM, Mutharasu G, Jayasinghe RG, Cao S, . Driver fusions and their implications in the development and treatment of human cancers. Cell Rep 2018;23:227–38.e3. 29617662 10.1016/j.celrep.2018.03.050 PMC5916809

[10] Heyer EE , Deveson IW, Wooi D, Selinger CI, Lyons RJ, Hayes VM, . Diagnosis of fusion genes using targeted RNA sequencing. Nat Commun 2019;10:1388. 30918253 10.1038/s41467-019-09374-9 PMC6437215

[11] Owen D , Ben-Shachar R, Feliciano J, Gai L, Beauchamp KA, Rivers Z, . Actionable structural variant detection via RNA-NGS and DNA-NGS in patients with advanced non-small cell lung cancer. JAMA Netw Open 2024;7:e2442970. 39495511 10.1001/jamanetworkopen.2024.42970 PMC11536281

[12] Passaro A , Al Bakir M, Hamilton EG, Diehn M, André F, Roy-Chowdhuri S, . Cancer biomarkers: emerging trends and clinical implications for personalized treatment. Cell 2024;187:1617–35. 38552610 10.1016/j.cell.2024.02.041 PMC7616034

[13] He X , Jiao X-D, Liu K, Qin B-D, Wu Y, Ling Y, . Clinical responses to crizotinib, alectinib, and lorlatinib in a metastatic colorectal carcinoma patient with ALK gene rearrangement: a case report. JCO Precis Oncol 2021;5:PO.20.00534. 34036227 10.1200/PO.20.00534 PMC8140796

[14] Takeyasu Y , Okuma HS, Kojima Y, Nishikawa T, Tanioka M, Sudo K, . Impact of ALK inhibitors in patients with ALK-rearranged nonlung solid tumors. JCO Precis Oncol 2021;5:PO.20.00383. 34036223 10.1200/PO.20.00383 PMC8140781

[15] Shreenivas A , Janku F, Gouda MA, Chen H-Z, George B, Kato S, . ALK fusions in the pan-cancer setting: another tumor-agnostic target? NPJ Precis Oncol 2023;7:101. 37773318 10.1038/s41698-023-00449-x PMC10542332

[16] Akhoundova D , Hussung S, Sivakumar S, Töpfer A, Rechsteiner M, Kahraman A, . ROS1 genomic rearrangements are rare actionable drivers in microsatellite stable colorectal cancer. Int J Cancer 2022;151:2161–71. 36053834 10.1002/ijc.34257 PMC9804412

[17] Hsiao S-Y , He H-L, Weng T-S, Lin C-Y, Chao C-M, Huang W-T, . Colorectal cancer with EML4-ALK fusion gene response to alectinib: a case report and review of the literature. Case Rep Oncol 2021;14:232–8. 33776709 10.1159/000511069 PMC7983623

[18] Subbiah V , Cassier PA, Siena S, Garralda E, Paz-Ares L, Garrido P, . Pan-cancer efficacy of pralsetinib in patients with RET fusion-positive solid tumors from the phase 1/2 ARROW trial. Nat Med 2022;28:1640–5. 35962206 10.1038/s41591-022-01931-y PMC9388374

[19] Drilon A , Laetsch TW, Kummar S, DuBois SG, Lassen UN, Demetri GD, . Efficacy of larotrectinib in TRK fusion-positive cancers in adults and children. N Engl J Med 2018;378:731–9. 29466156 10.1056/NEJMoa1714448 PMC5857389

[20] Beaubier N , Tell R, Lau D, Parsons JR, Bush S, Perera J, . Clinical validation of the tempus xT next-generation targeted oncology sequencing assay. Oncotarget 2019;10:2384–96. 31040929 10.18632/oncotarget.26797 PMC6481324

[21] Beaubier N , Bontrager M, Huether R, Igartua C, Lau D, Tell R, . Integrated genomic profiling expands clinical options for patients with cancer. Nat Biotechnol 2019;37:1351–60. 31570899 10.1038/s41587-019-0259-z

[22] Michuda J , Breschi A, Kapilivsky J, Manghnani K, McCarter C, Hockenberry AJ, . Validation of a transcriptome-based assay for classifying cancers of unknown primary origin. Mol Diagn Ther 2023;27:499–511. 37099070 10.1007/s40291-023-00650-5 PMC10300170

[23] Bray NL , Pimentel H, Melsted P, Pachter L. Near-optimal probabilistic RNA-seq quantification. Nat Biotechnol 2016;34:525–7. 27043002 10.1038/nbt.3519

[24] Kuznetsova A , Brockhoff PB, Christensen RHB. LmerTest package: tests in linear mixed effects models. J Stat Softw 2017;82:1–26.

[25] Benayed R , Offin M, Mullaney K, Sukhadia P, Rios K, Desmeules P, . High yield of RNA sequencing for targetable kinase fusions in lung adenocarcinomas with no mitogenic driver alteration detected by DNA sequencing and low tumor mutation burden. Clin Cancer Res 2019;25:4712–22. 31028088 10.1158/1078-0432.CCR-19-0225 PMC6679790

[26] Laskin J , Liu SV, Tolba K, Heining C, Schlenk RF, Cheema P, . NRG1 fusion-driven tumors: biology, detection, and the therapeutic role of afatinib and other ErbB-targeting agents. Ann Oncol 2020;31:1693–703. 32916265 10.1016/j.annonc.2020.08.2335 PMC8911318

[27] Gupta B , Gosa Barrett L, Liu SV. NRG1 fusions in NSCLC: being eNRGy conscious. Lung Cancer (Auckl) 2024;15:143–8. 39376790 10.2147/LCTT.S464626 PMC11457762

[28] Yang Q , Wang B, Meng X, Ma T, Qian Z, Cai X, . Unveiling the BRAF fusion structure variations through DNA and RNA sequencing. Br J Cancer 2025;132:1177–87. 40253487 10.1038/s41416-025-02998-3 PMC12152160

[29] Hackshaw A , Fajardo O, Dafni U, Gelderblom H, Garrido P, Siena S, . Characteristics and survival outcomes of patients with metastatic RET fusion-positive solid tumors receiving non-RET inhibitor standards of care in a real-world setting. JCO Precis Oncol 2024;8:e2300334. 38271655 10.1200/PO.23.00334 PMC10830092

[30] Desilets A , Repetto M, Yang S-R, Sherman EJ, Drilon A. RET-altered cancers-a tumor-agnostic review of biology, diagnosis and targeted therapy activity. Cancers (Basel) 2023;15:4146. 37627175 10.3390/cancers15164146 PMC10452615

[31] Han S-Y . TRK inhibitors: tissue-agnostic anti-cancer drugs. Pharmaceuticals (Basel) 2021;14:632. 34209967 10.3390/ph14070632 PMC8308490

[32] Schram AM , Goto K, Kim D-W, Macarulla T, Hollebecque A, O’Reilly EM, . Efficacy of zenocutuzumab in NRG1 fusion-positive cancer. N Engl J Med 2025;392:566–76. 39908431 10.1056/NEJMoa2405008 PMC11878197

[33] Center for Drug Evaluation, Research . FDA grants accelerated approval to zenocutuzumab-zbco for non-small cell lung cancer and pancreatic adenocarcinoma [Internet]. Silver Spring (MD): U.S. Food and Drug Administration. FDA; 2024 [cited 2025 Apr 25]. Available from: https://www.fda.gov/drugs/resources-information-approved-drugs/fda-grants-accelerated-approval-zenocutuzumab-zbco-non-small-cell-lung-cancer-and-pancreatic.

[34] Westphalen CB , Krebs MG, Le Tourneau C, Sokol ES, Maund SL, Wilson TR, . Genomic context of NTRK1/2/3 fusion-positive tumours from a large real-world population. NPJ Precis Oncol 2021;5:69. 34285332 10.1038/s41698-021-00206-y PMC8292342

[35] Ross JS , Wang K, Chmielecki J, Gay L, Johnson A, Chudnovsky J, . The distribution of BRAF gene fusions in solid tumors and response to targeted therapy. Int J Cancer 2016;138:881–90. 26314551 10.1002/ijc.29825 PMC5049644

[36] Parimi V , Tolba K, Danziger N, Kuang Z, Sun D, Lin DI, . Genomic landscape of 891 RET fusions detected across diverse solid tumor types. NPJ Precis Oncol 2023;7:10. 36690680 10.1038/s41698-023-00347-2 PMC9870857

[37] Jonna S , Feldman RA, Swensen J, Gatalica Z, Korn WM, Borghaei H, . Detection of NRG1 gene fusions in solid tumors. Clin Cancer Res 2019;25:4966–72. 30988082 10.1158/1078-0432.CCR-19-0160 PMC7470623

[38] Bruno R , Fontanini G. Next generation sequencing for gene fusion analysis in lung cancer: a literature review. Diagnostics (Basel) 2020;10:521. 32726941 10.3390/diagnostics10080521 PMC7460167

[39] Ramani NS , Patel KP, Routbort MJ, Alvarez H, Broaddus R, Chen H, . Factors impacting clinically relevant RNA fusion assays using next-generation sequencing. Arch Pathol Lab Med 2021;145:1405–12. 33493304 10.5858/arpa.2020-0415-OA

[40] Finall A . RNA-based next-generation sequencing in the somatic molecular testing of non-small-cell lung cancer (NSCLC) in a centralized model: real-world data to suggest it is time to reconsider testing options. J Mol Pathol 2022;3:307–18.

[41] De Luca C , Pepe F, Iaccarino A, Pisapia P, Righi L, Listì A, . RNA-based assay for next-generation sequencing of clinically relevant gene fusions in non-small cell lung cancer. Cancers (Basel) 2021;13:139. 33406752 10.3390/cancers13010139 PMC7796105

