# Supplementary material for: Molecular Characterization of Oncogenic Gene Fusions in a Large Real-World Cohort of Solid Tumors
Source: Cancer Res Commun. 2025 Nov 6;5(11):1967–76. doi: 10.1158/2767-9764.CRC-25-0329 (PMC12589939; doi:10.1158/2767-9764.CRC-25-0329)
Supplement: Supplementary Text — S1 [file crc-25-0329_supplementary_text_suppst1.pdf]

**Supplementary Text S1:**

**xR Analytical Validation - Limit of Blank**

**Goal of Study:** Determine the limit of blank (LOB)—the threshold of total supporting reads at which a negative call is confidently called—for Tempus xR.

**Methods:** Specifically, the limit of blank for fusions was evaluated by testing wildtype clinical FFPE samples and determining the false positive rate over multiple runs split over 3 days and 2 reagent lots. All blank sample measurements were sorted from lowest to highest using the maximum gene rearrangement normalized total support for any gene pair. The 95th percentile was calculated for the distribution of blank sample results (PctB) corresponding to type I error risk probability $\alpha$=0.05. The rank position corresponding to the PctB percentile was calculated as: Rank position = 0.5 +B * 0.95 where B is the total number of results in the dataset.

**Samples:** All samples originated from FFPE specimens expected to be wild type for gene fusions with tumor purities ranging from 90% down to 30%. 24 unique samples were tested in duplicate using two different reagent lots (n=4 per sample) for a total of 96 measurements (**Supplementary Table S2).**

**Results:** All samples other than one glioblastoma sample had 0 total supporting reads detected for reportable fusions across all lot numbers. The glioblastoma sample had 3 total supporting reads for fusion VOPP1-EGFR. Confirmatory testing with dPCR QIAcuity One showed inconclusive/low positive dPCR results (2 replicates below dPCR threshold, and 4 replicates above dPCR threshold). Since dPCR is expected to be more sensitive than NGS, and results were inconsistent, a conservative approach was used to treat VOPP1-EGFR as a true false positive by NGS in this study and adjust the NGS LOB threshold accordingly.

The LOB threshold was set at 3 total supporting reads for fusions with ≥4 total reads required to call a positive fusion.

**xR Analytical Validation - Fusion Accuracy**

**Goal of Study:** Report the accuracy of Fusions compared to an orthogonal method.

**Acceptance Criteria Details:** The device shall show ≥90% point estimate of PPA and NPA for detection of fusions, with the lower bound of 95% CI ≥90%, when compared to a valid orthogonal method.

**Methods:** The ability of the Tempus xR assay to accurately detect gene fusions was evaluated by comparing results from Tempus xR to results from a validated orthogonal method. The orthogonal test method used in this study was the FusionPlex BBI Solid Tumor Panel performed at University of Washington Clinical Genomics Laboratory (Seattle, WA), a CAP/CLIA certified laboratory. The FusionPlex panel uses Anchored Multiplex PCR (AMP) chemistry (Archer Dx, CO) followed by next generation sequencing and targets 114 genes commonly mutated in solid tumor type cancers. The orthogonal test is a targeted NGS panel that requires a minimum of 5

unique reads for fusion detection. Positive fusion detection for the xR assay was set as 4 or more total reads based on the LOB study described above. Fusions assessed for accuracy must have genes called on the xR assay that overlap with the orthogonal method.

Agreement was calculated at the variant level. PPA was calculated as 100*(overlapping test reportable positives from both methods/non ref. std. reportable positives). NPA was calculated as 100*(overlapping test reportable negatives from both methods/non ref. std. reportable negatives). False negatives were calculated as total number of samples x 52 possible gene fusion pairs outcomes/sample - number of true positives - number of false negatives.

**Samples:** Specimens used in this study originated from RNA derived from FFPE tumor tissue. 290 unique samples were evaluated in this study. Samples originated from 28 different tumor types (**Supplementary Table S3**). All samples had an RNA mass input of 100 ng except for 3 samples which had a mass input of 50 ng and 7 samples which had a mass input of 300 ng. Tumor purity ranged from 20%-90%.

**Results:**
Using the orthogonal method as the source of truth, 168 fusions were true positives in 165 samples. One false positive and three false negatives were identified in four samples. The discordant samples are described in **Supplementary Table S4.** In 122 samples, 14,908 fusions were true negatives. PPA was 98.2% (95% CI 94.97%, 99.40%) and NPA was 99.993% (95% CI 99.96%,≥ 99.99%), satisfying the acceptance criteria of the study. Samples and concordance are shown in **Supplementary Table S3.**
