# Supplementary material for: Molecular Characterization of Oncogenic Gene Fusions in a Large Real-World Cohort of Solid Tumors
Source: Cancer Res Commun. 2025 Nov 6;5(11):1967–76. doi: 10.1158/2767-9764.CRC-25-0329 (PMC12589939; doi:10.1158/2767-9764.CRC-25-0329)
Supplement: Supplementary Figure 1 — Supplemental Figure 1 [file crc-25-0329_supplementary_figure_1_suppsf1.pdf]

Supplementary Figure 1.

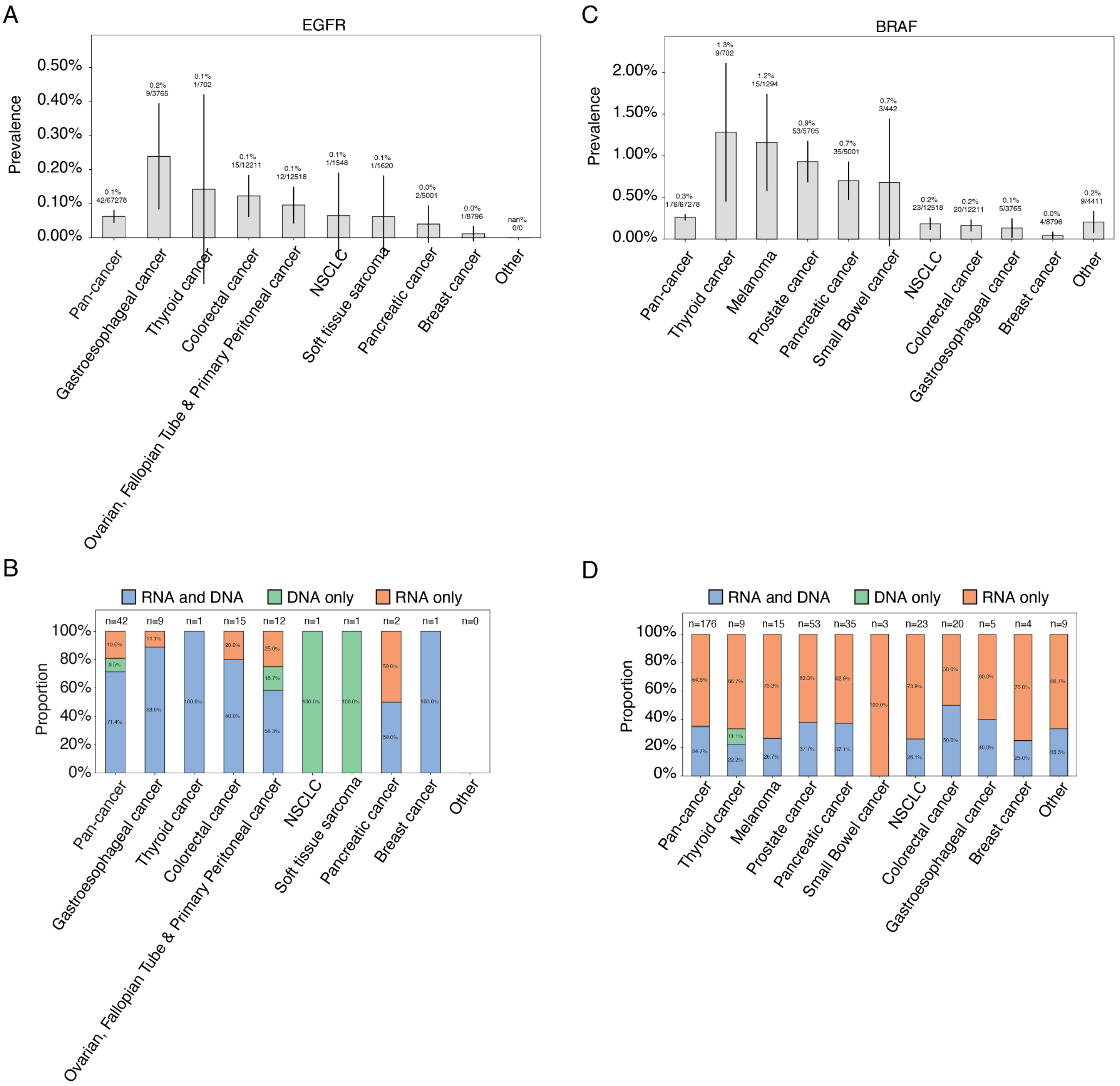

Figure S1: a) Fusion prevalence for EGFR by cancer type. b) The percent of EGFR driver fusions detected by analyte: RNA-only, DNA-only, or detected by both RNA and DNA. c) Fusion prevalence for BRAF by cancer type. b) The percent of BRAF driver fusions detected by analyte: RNA-only, DNA-only, or detected by both RNA and DNA. For all sub-plots, cancer types with < 500 patients were aggregated into the “Other” category.
